# Left ventricular thrombus with extracorporeal membrane oxygenation: Novel technique of bronchoscope-guided thrombus retrieval

**DOI:** 10.1016/j.xjtc.2022.08.008

**Published:** 2022-08-13

**Authors:** Jennifer L. Perri, Georg M. Wieselthaler

**Affiliations:** Division of Adult Cardiothoracic Surgery, University of California San Francisco, San Francisco, Calif

**Figure:**
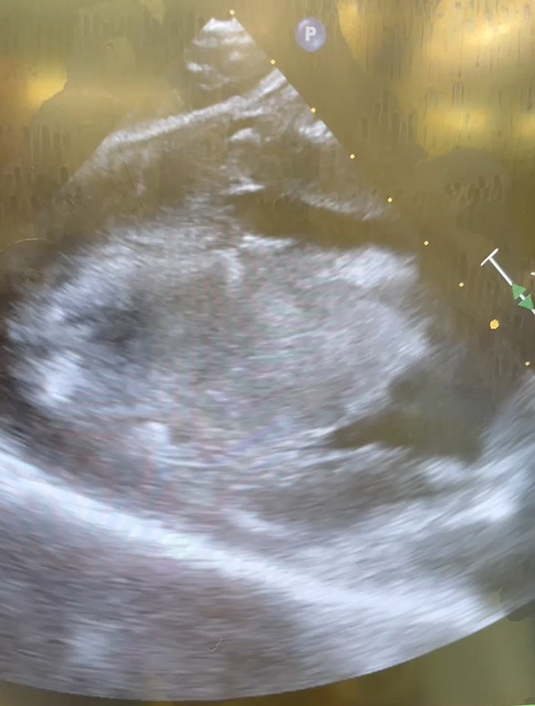
Thrombus obliterates the left ventricle; a flexible bronchoscope improves visualization for retrieval.

**Central Message**

A flexible bronchoscope is used as an adjunct to retrieve LV thrombus through an aortotomy; the scope improves visualization and ease of clot retrieval.

Every year approximately 2500 patients in the United States receive venoarterial (VA) extracorporeal membrane oxygenation (ECMO) for cardiogenic shock.^1^ In cases of low contractility, intraventricular stasis, or inadequate anticoagulation, patients are prone to developing intracardiac thrombus. In the largest study to date on rates of thrombus formation during peripheral VA ECMO, of 281 patients in a tertiary care center, 4% had thrombus formation, and in all cases this eventually resulted in in-hospital mortality.^2^ Herein we present a case and a reproducible technique for quickly removing intracardiac thrombus.

The institutional review board or equivalent ethics committee of the University of California San Francisco Medical Center did not approve this study because this is a case report on a single case, and therefore is institutional review board exempt. The patient provided informed written consent for the publication of the study data.

## Case

**Figure 1:**
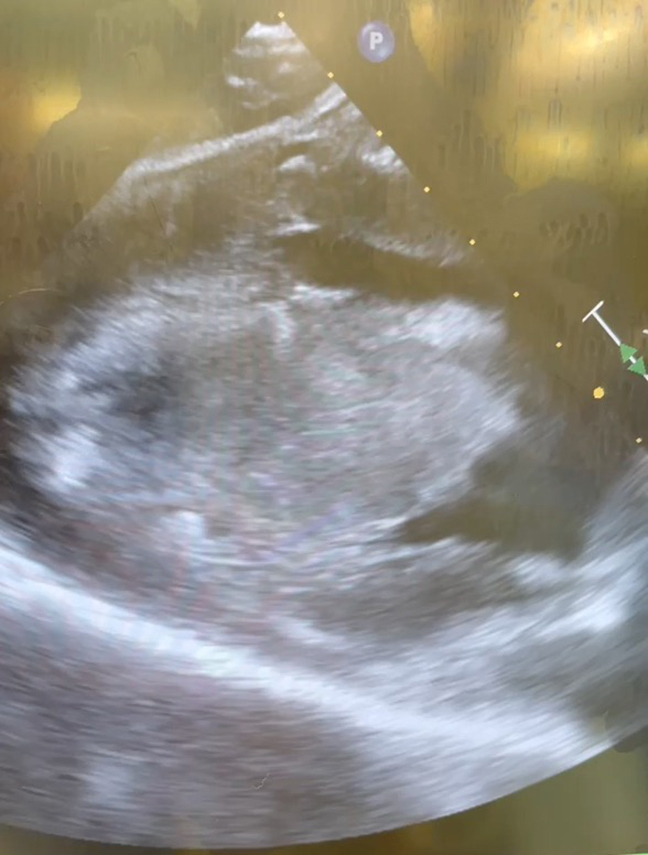
Thrombus obliterates the left ventricle; a flexible bronchoscope improves visualization for retrieval.

A 23-year-old male patient with history of mechanical aortic and mitral valve replacements in India presented in severe cardiogenic shock with massive pulmonary edema. The patient tested positive for COVID-19 and was given femoral VA ECMO. Echocardiogram revealed an ejection fraction of 10% to 15%, thrombosed aortic prosthesis, and severe aortic regurgitation. The patient underwent a redo sternotomy and complex placement of a biological 21-mm Trifecta aortic valve prosthesis (Abbott). Surgery was notable for diffuse adhesions and excessive bleeding; hence a tissue valve was placed. At case end, the patient continued to receive VA ECMO and a left atrial vent (15-French heparin coated cannula) was placed to reduce the pulmonary edema, and offload the left side of the heart. The patient was brought back to the operating room on postoperative day (POD) 2, by which time pulmonary edema had resolved. Despite a heparin drip with activated partial thromboplastin time of 45 to 55, the transesophageal echocardiogram revealed thrombus formation obliterating the left ventricular (LV) cavity (Figure 1). The aorta was crossclamped and large portions of the thrombus were removed with grasping instruments. However, significant thrombus remained in the apex on postoperative echocardiogram; the apex was inaccessible because of poor visualization and the position of the heart with preexisting adhesions. Intracardiac thrombus was subsequently removed under direct vision with a flexible bronchoscope.

## Surgical Technique

**Figure 2:**
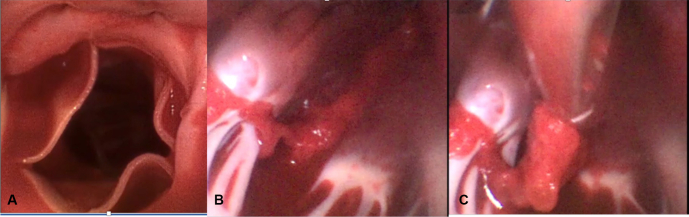
Flexible bronchoscope provides excellent visualization on entry through the aortic valve (A), of clot adherent to the trabecula in the left ventricle (B), allowing for easy retrieval with a minimally invasive forceps grasper (C).

After a horizontal aortotomy a large single-use flexible bronchoscope (4 Broncho Regular 5.0/2.2; Ambu aScope) was passed beyond the prosthetic aortic valve into the apex of the left ventricle. Using a combination of long, straight grasping instruments, and a toothed biopsy forceps placed through the working canal of the scope, the entire thrombus was removed under vision (Figure 2). Crossclamp time was 79 minutes. Video 1 illustrates ease of retrieval.

The patient was decannulated from VA ECMO on POD 13, and discharged to home on POD 41. His ejection fraction improved from 10% to 15% at admission to 65% at discharge.

## Discussion

Patients with severely reduced contractility and concomitant stasis in the left ventricle are prone to developing thrombus; in this case a left atrial vent and low levels of anticoagulation in the setting of postoperative bleeding further increased risk of thrombus formation. Thrombus, when it develops in the left ventricle, is approached in 1 of 2 ways—through the left atrium and the mitral valve, or the ventricular wall itself. A transmitral approach was not possible because an existing mechanical prosthesis was in place. The second option via an open ventriculotomy would have negatively affected contractility of an already failing heart. Therefore, the transaortic approach offers value in preserving LV function, but the downside is limited visualization of the entire intracardiac cavity and restricted manipulation using long, straight instruments. The practice of using a videoscope from within a cardiac chamber is termed “cardioscopy.”^3^ Case reports as early as 1999 describe use of a straight videoscope (5-mm or 10-mm rigid scope).^4^^,^^5^ With a redo sternotomy and dense adhesions, rigid instruments were not adequate in this case. A flexible bronchoscope allows 360° rotation, advantageous in a redo setting. Of 36 reports on scope-guided LV mass removal, only 3 report on flexible endoscopes.^6^ Compared with an endoscope such as that for gastrointestinal procedures, the bronchoscope is less cumbersome and more readily available in a cardiothoracic operating arena.

LV thrombus is a known occurrence in patients with poor contractility during ECMO. Extracorporeal Life Support Organization guidelines advise use of a plasma-based test (eg, activated partial thromboplastin time, anti-Xa) and whole blood test (eg, activated clotting time, thromboelastography/rotational thromboelastography) to monitor anticoagulation. Thromboelastography/rotational thromboelastography are used as point of care testing to guide blood product administration but testing in ECMO patients for thrombotic events is not yet proven.^7^ Regular echocardiogram, improvement in monitoring of anticoagulation,^7^ and an efficient method of thrombus removal are of paramount importance. Our experience suggests transaortic removal of LV thrombus with visual guidance from a sterile, flexible bronchoscope, not the typical approach, is reproducible, efficient, and effective.

## Supplementary Data

Video 1 Description of case with intraoperative video. Video available at: https://www.jtcvs.org/article/S2666-2507(22)00445-X/fulltext.
