# Supplementary figures and images for: Left ventricular thrombus with extracorporeal membrane oxygenation: Novel technique of bronchoscope-guided thrombus retrieval
Source: JTCVS Tech. 2022 Aug 13;15:130–2. doi: 10.1016/j.xjtc.2022.08.008 (PMC9579871; doi:10.1016/j.xjtc.2022.08.008)

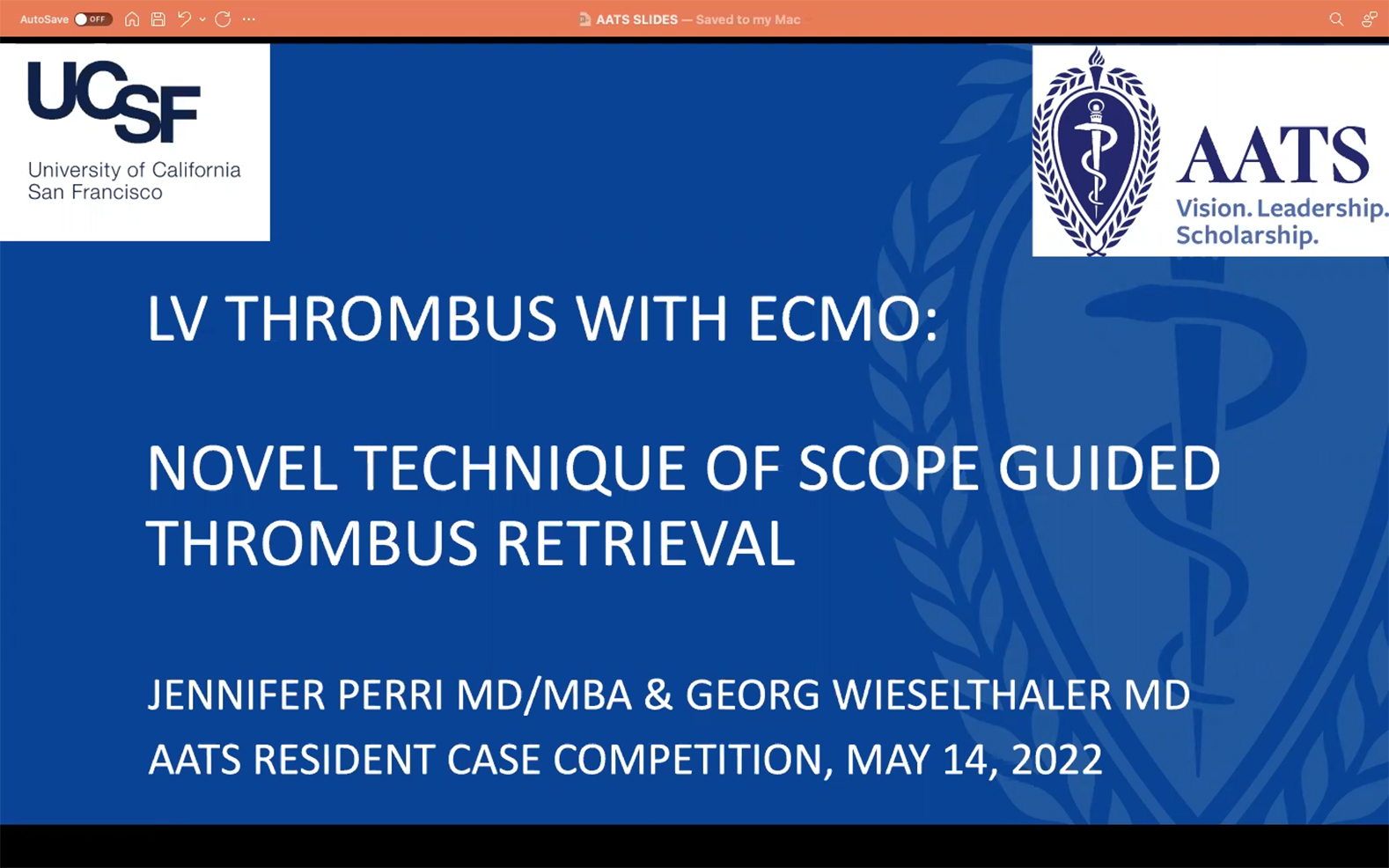

Supplement: Video 1 — Description of case with intraoperative video. Video available at: https://www.jtcvs.org/article/S2666-2507(22)00445-X/fulltext. [file fx2.jpg]
